# Motivational Strategies for Stroke Rehabilitation: A Descriptive Cross-Sectional Study

**DOI:** 10.3389/fneur.2020.00553

**Published:** 2020-06-10

**Authors:** Kazuaki Oyake, Makoto Suzuki, Yohei Otaka, Satoshi Tanaka

**Affiliations:** ^1^Department of Physical Therapy, School of Health Sciences, Shinshu University, Nagano, Japan; ^2^Faculty of Health Sciences, Tokyo Kasei University, Saitama, Japan; ^3^Department of Rehabilitation Medicine I, School of Medicine, Fujita Health University, Aichi, Japan; ^4^Laboratory of Psychology, Hamamatsu University School of Medicine, Shizuoka, Japan

**Keywords:** cerebrovascular disease, cluster analysis, motivation, motivational model, strategy

## Abstract

**Background:** The addition of motivational strategies to a rehabilitation program is thought to enhance patient adherence and improve outcomes. However, little is known about how rehabilitation professionals motivate stroke patients during rehabilitation. The primary objective of this study was to provide a comprehensive and quantitative list of motivational strategies for stroke rehabilitation. In addition, we aimed to examine (1) whether professionals with more clinical experience used a higher number of motivational strategies, (2) the purpose for using each strategy, and (3) the information considered when choosing strategies.

**Methods:** This descriptive, cross-sectional study was conducted using a web survey with a convenience sample of 407 rehabilitation professionals including physicians, nurses, physical therapists, occupational therapists, and speech-language-hearing therapists.

**Results:** We received data for 362 participants. Fifteen strategies were found to be used by more than 75% of the respondents to motivate their patients. Almost all of the respondents reported that they actively listened to and praised their patients to increase patient adherence to rehabilitation programs. Respondents with more clinical experience tended to use a higher number of motivational strategies (rho = 0.208, *p* < 0.001). For 11 of the 15 strategies selected by more than 75% of the respondents, the highest percentage of respondents reported that they used the strategies to make rehabilitation worthwhile for their patients. The majority of respondents reported that they decided which motivational strategy to use by considering comprehensive information regarding the patient health condition, environmental factors, and personal factors.

**Conclusions:** The comprehensive list of motivational strategies obtained may be useful for increasing patient adherence to rehabilitation, especially for professionals with less clinical experience. Furthermore, our findings regarding the purpose for using each strategy and the information considered when choose strategies might help rehabilitation professionals to optimally utilize the motivational strategy list.

## Introduction

Studies on stroke rehabilitation have recommended the use of intensive and repetitive task-specific practice, as well as aerobic exercise (1). Given that the independent efforts of the patient are necessary to sustain these practices and exercises, patient motivation is frequently used as a determinant of rehabilitation outcome (2). High adherence to a rehabilitation program is thought to be indicative of motivation (2, 3), and a lack of motivation is one of the perceived barriers to physical activity and exercise training after stroke (4–7). Therefore, the addition of motivational strategies to rehabilitation programs may effectively enhance patient adherence, producing better outcomes (8).

Motivational strategies such as feedback (9, 10), counseling (11), and information provision (12) have positive effects on recovery after stroke. An international randomized clinical trial found that praise and positive feedback were effective for improving walking speed during inpatient rehabilitation (9). Feedback using virtual reality has been found to be beneficial in improving motivation, upper limb function, and activities of daily living (10, 13). Furthermore, counseling and information provision have been found to have a positive impact on mood (11, 12). However, few reports have comprehensively investigated strategies used by medical professionals to motivate patients undergoing stroke rehabilitation.

Maclean et al. (3) conducted a semi-structured interview of medical professionals to determine how they increase patient motivation with respect to stroke rehabilitation. The researchers reported that setting rehabilitation goals, providing information about rehabilitation, and accessing and using the patient's cultural norms appeared to have a positive effect on motivation (3). However, it is difficult to generalize these findings to the general population due to the small sample size and in-depth interview method used (14).

As opposed to semi-structured interviews, the findings from quantitative surveys are generalizable to a larger population (14). Therefore, the primary objective of this study was to provide a comprehensive list of motivational strategies that medical professionals use for stroke rehabilitation in Japan based on quantitative survey data. A list of motivational strategies is likely to be useful in increasing patient adherence to rehabilitation programs. Based on our clinical experience, we hypothesized that motivational skills could be acquired through clinical experience. In addition, understanding the purpose of each motivational strategy and the information that is evaluated when choosing strategies may contribute to effective utilization of the list. Thus, our secondary objectives were to examine (1) whether rehabilitation professionals with greater clinical experience used more motivational strategies, (2) the purpose for using each strategy, and (3) the information considered when choosing motivational strategies.

## Materials and Methods

### Study Design

This study had a descriptive cross-sectional design. We used a convenience sampling web-based survey to obtain quantitative results from the participant perspective. The study protocol was approved by the appropriate ethics committee at the Hamamatsu University School of Medicine (approval number: 18-136). Informed consent was obtained from all participants.

### Participants

Eligible participants were rehabilitation professionals including physicians, nurses, physical therapists, occupational therapists, speech-language-hearing therapists, or clinical psychologists currently working in rehabilitation. Participants were recruited with the cooperation of the 33rd conference of the Comprehensive Rehabilitation Ward Association and the 48th Annual Meeting of the Nagano Physical Therapy Association. To recruit participants, we set up a booth with laptops at each of the above-mentioned conferences. Professionals who were interested in participating in the study were able to access the survey website using the provided laptops. In addition, we distributed leaflets to professionals and displayed posters containing a brief description of the study and a hyperlink to the survey in front of the booth. Respondents were able to access the survey website using their own laptops, tablets, or smartphones. The first page of the survey informed participants about the total number of questions, the approximate time required to complete the survey, and the aim of the study. Participants were asked to report their professional category and years of experience working in stroke rehabilitation. Those who met the eligible criteria proceeded to the next page.

### Survey Instrument

We consulted existing guidelines and followed a checklist when preparing the survey (15, 16). We chose to use a voluntarily accessed survey format developed using the Google Forms tool (Google LLC, Mountain View, CA, USA). Among the individuals who completed the survey, 50 were selected via a draw to receive an honorarium of 1,000 yen (~US $9.00).

The survey items regarding motivational strategies were developed based on the clinical experience of the authors, the findings of related literature (2–5, 7, 9, 11, 12, 17–20), and data obtained from semi-structured interviews with five professionals about motivational strategies for stroke rehabilitation (21). Stroke rehabilitation experts were asked to review the items for clarity, relevance, and topic coverage (22). We carried out a pilot test with a group of 10 rehabilitation professionals to determine whether the respondents understood the questions and instructions, and whether the meaning of the questions was the same for all respondents (16). The survey took an average of 15 min to complete. According to the qualitative feedback obtained from the semi-structured interviews with the 10 respondents, we made some minor grammatical changes to the survey. Consequently, we prepared a list of 22 motivational strategies (Table 1). In the first section of the survey, respondents were shown the list of motivational strategies and asked if they used each strategy in their clinical practice. Respondents were also asked to respond to an open-ended question at which point they were invited to propose additional motivational strategies that were not included in the list.

**Table 1:** List of motivational strategies.

**Motivational strategy**	**Representation in the manuscript**
Active listening	Active listening
Allowing the patient to use a newly acquired skill	Allowing the patient to use a newly acquired skill
Applying patient preferences to practice and exercise tasks	Application of patient's preferences
Control of task difficulty	Control of task difficulty
Explaining the necessity of a practice	Explaining the necessity of a practice
Goal-oriented practice	Goal-oriented practice
Goal setting	Goal setting
Group rehabilitation	Group rehabilitation
Engaging in enjoyable communication with the patient	Enjoyable communication
Providing the patient positive evaluation and encouragement	Praise
Proposing conditions for exchange (e.g., promising to undertake the patient's favorite practice after achieving his/her least favorite practice)	Proposing conditions for exchange
Providing a suitable rehabilitation environment	Providing a suitable rehabilitation environment
Providing exercise and practice with game properties	Practice with game properties
Providing medical information	Providing medical information
Providing opportunities for the patient to identify possible treatments	Providing opportunities to identify possible treatments
Providing practice tasks relating to the patient's experience and lifestyle	Practice related to patient's experience
Providing variations of rehabilitation programs to sustain interest	Providing variations of the program
Recommending the presence of a family member during rehabilitation	Family member present
Respect for self-determination	Respect for self-determination
Sharing the criteria for evaluation	Sharing the criteria for evaluation
Specifying the amount of practice and exercise that will be required	Specifying the amount of practice required
Using tools such as a diary or graph that enables the patient to track his/her progress	Using progress-confirming tools

*Motivational strategies are arranged in alphabetical order*.

In the second section, for each motivational strategy that a respondent reported using, they were asked to state their aim when using that strategy. Specifically, they were asked to select from the following four purposes: (1) to increase the patient's interest in rehabilitation; (2) to make rehabilitation more worthwhile for the patient; (3) to help the patient gain confidence in performing a rehabilitation task; and (4) to increase patient satisfaction with the rehabilitation program. These purposes were based on the four sub-components of motivation proposed by the Attention, Relevance, Confidence, and Satisfaction (ARCS) model (23, 24). The ARCS model is a problem-solving approach that is used when designing motivational aspects of learning environments with the goal of stimulating and sustaining students' motivation to learn (23). In the third section, respondents were shown 11 items related to the patient health status, environmental factors, and personal factors (Table 2) (25). They were asked to select all of the items that they considered when deciding which motivational strategy to use. Finally, respondents were asked to report their gender, primary affiliation, and the phase of stroke recovery of the patients with which they most frequently worked.

**Table 2:** Information used when determining how to motivate a patient with stroke.

**Information**
Cognitive function (e.g., higher brain dysfunction and dementia)
Comorbidities (e.g., psychological disorder, diabetes mellitus, and infection)
Demographic characteristics such as age and sex
Diagnosis (e.g., ischemic or hemorrhagic, lesion site, and recurrence)
Human environment (e.g., key person and family)
Patient's reaction to a presented motivational strategy
Personality
Physical function (e.g., muscle weakness, limited range of motion, and sensory dysfunction)
Severity of activity limitations
Severity of participation restrictions
Social environment (e.g., economic condition and employment status)

*Information arranged in alphabetical order*.

### Sample Size

We used the Cochran sample size formula to calculate the sample size. We set an alpha level a priori at 0.05, an acceptable error level of 5%, and a confidence interval of 95% (26). Consequently, a minimum of 384 participants were required. Assuming that the data from approximately 5% of the participants would be excluded from analysis, we aimed to recruit a total of 400 participants.

### Statistical Analysis

We used descriptive statistics to characterize the study sample and summarize the participant responses. The normality of distribution was tested using the Shapiro-Wilk test. We used Pearson's product-moment correlation coefficient or Spearman's rank correlation coefficient to test whether respondents with more experience used more motivational strategies. In addition, we used a stepwise multiple regression analysis to examine the relationship between the years of clinical experience and the number of motivational strategies used by each respondent, while controlling for potentially confounding variables. We included the respondents' gender, professional category, primary affiliation, and the patient phase of stroke recovery in the model as covariates. Dummy variables were used to incorporate categorical variables such as gender and professional category into the regression model. For the motivational strategies that were used by at least 75% of respondents (27), we conducted a hierarchical cluster analysis using Ward's method with a squared Euclidean distance to group them according to the purpose of use. Statistical analyses were performed using the Statistical Package for the Social Sciences software version 25.0 (International Business Machines Corp., NY, USA). Any *p* values < 0.05 were considered statistically significant.

### Reporting

This study was reported according to the recommended best practices and guidelines in the literature for the reporting of survey research (16, 22).

## Results

**Figure 1:**
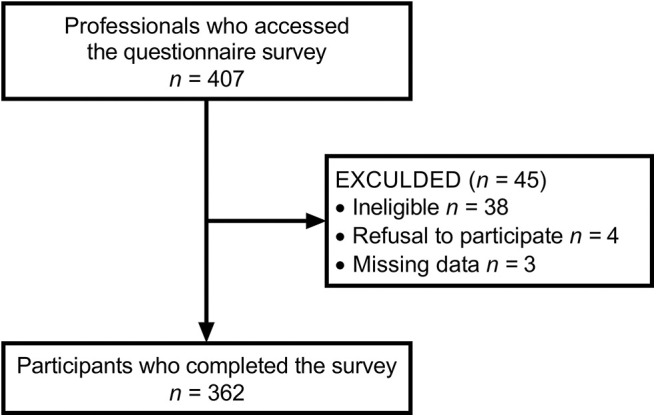
Flow diagram of the participant selection process.

The survey was conducted from February to July 2019. Approximately 4,150 rehabilitation professionals attended the two conferences. Among them, 407 professionals accessed the survey. Therefore, the response rate was ~9.8%. Forty-five respondents were excluded because they did not meet the eligibility criteria, withdrew consent to participate, or had missing data. Consequently, 362 respondents completed the survey. The flow of participants is shown in Figure 1. Table 3 shows the characteristics of the respondents. The majority of the respondents were physical therapists (51.1%), had <5 years of clinical experience (36.2%), were female (53.0%), were employed in a hospital (91.2%), and worked with patients with subacute stroke (71.3%). No clinical psychologists participated in the survey.

**Table 3:** Respondent characteristics (*n* = 362).

**Variable**	**Value**
Professional category	
Physical therapist	185 (51.1)
Nurse	82 (22.7)
Occupational therapist	74 (20.4)
Speech-language-hearing therapist	13 (3.6)
Physician	8 (2.2)
Years of experience in stroke rehabilitation	
Less than 5 years	131 (36.2)
5–9 years	90 (24.8)
10–14 years	79 (21.8)
15–19 years	31 (8.6)
20 years or more	31 (8.6)
Sex	
Female	192 (53.0)
Male	170 (47.0)
Primary affiliation	
Hospital	333 (92.0)
Geriatric health services facility or nursing home	16 (4.4)
Others	13 (3.6)
Phase of stroke recovery of the patients	
Subacute	258 (71.2)
Chronic	46 (12.7)
Acute	44 (12.2)
Others	14 (3.9)

*Values are presented as number (%)*.

### Which Strategies Do Rehabilitation Professionals use to Motivate Their Patients?

**Figure 2:**
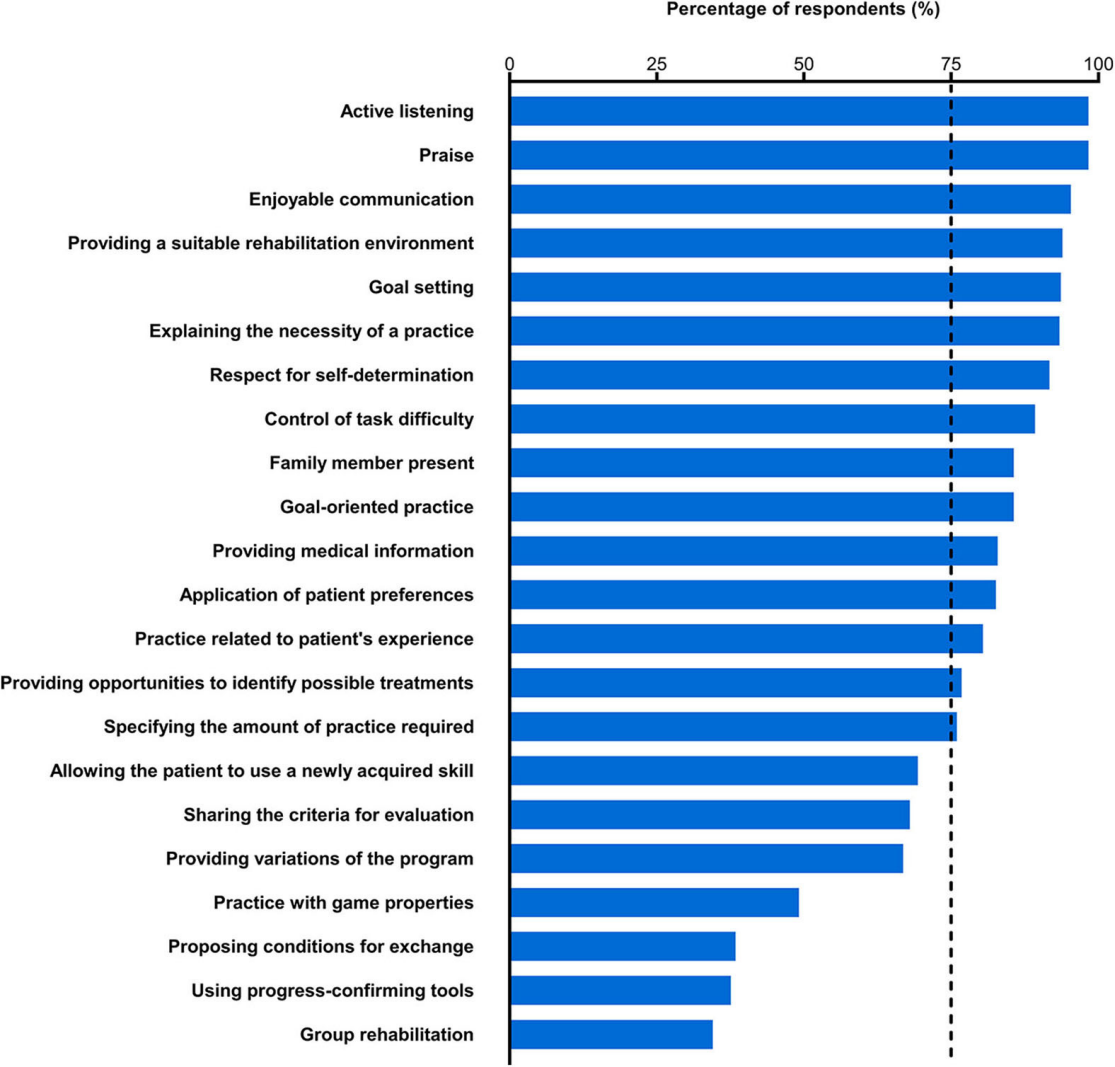
Percentage of respondents who reported that they used each presented motivational strategy during stroke rehabilitation. The vertical dashed line represents 75% of the respondents. The motivational strategies are arranged in descending order by the percentage of respondents who stated that they used each strategy.

**Figure 3:**
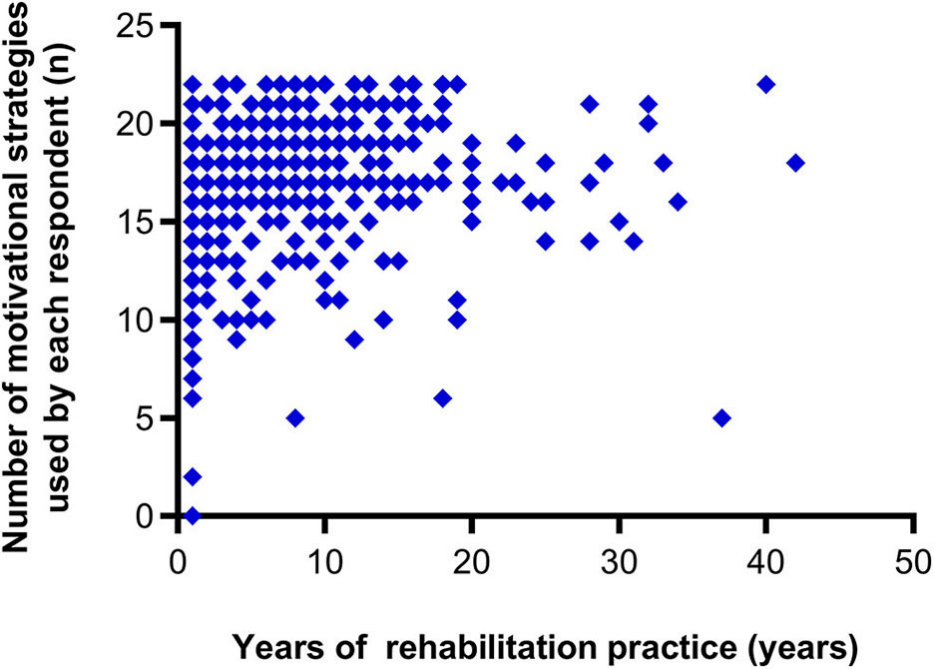
Correlation between the number of motivational strategies used by each respondent and years of rehabilitation practice.

The percentages of respondents who used each of the presented motivational strategies are shown in Figure 2. The majority of the respondents (98.3%) selected “active listening” and “praise.” “Enjoyable communication,” “providing a suitable rehabilitation environment,” “goal setting,” “explaining the necessity of a practice,” and “respect for self-determination” were also selected by more than 90% of the respondents (95.3–91.7%). The majority of the respondents reported that they used “control of task difficulty,” “family member present,” “goal-oriented practice,” “providing medical information,” “application of patient preferences,” “practice related to patient's experience,” “providing opportunities to identify possible treatments,” and “specifying the amount of practice required” (89.2–76.0%). Thus, 15 out of the 22 presented strategies were selected by more than 75% of the respondents. Between 69.3 and 66.9% of the respondents reported that they used “allowing the patient to use a newly acquired skill,” “sharing the criteria for evaluation,” and “providing variations of the program.” Less than half of the respondents selected “practice with game properties,” “proposing conditions for exchange,” “using progress-confirming tools,” and “group rehabilitation” (49.2–34.5%). The results by professional category are shown in Supplemental Figure 1. The numbers of motivational strategies selected by at least 75% of the respondents who were nurses, physical therapists, and occupational therapists were 10, 15, and 18, respectively. The respondents proposed four additional motivational strategies (Supplemental Table 1). Respondents with more clinical experience tended to use a larger number of motivational strategies (rho = 0.208, 95% confidence interval = 0.105, 0.308, *p* < 0.001) (Figure 3). In addition, even when controlling for potentially confounding variables, the relationship between years of clinical experience and the number of motivational strategies used by each respondent remained significant (*t* = 2.027, *p* = 0.043).

### For What Purpose Do Rehabilitation Professionals use Each Motivational Strategy?

**Figure 4:**
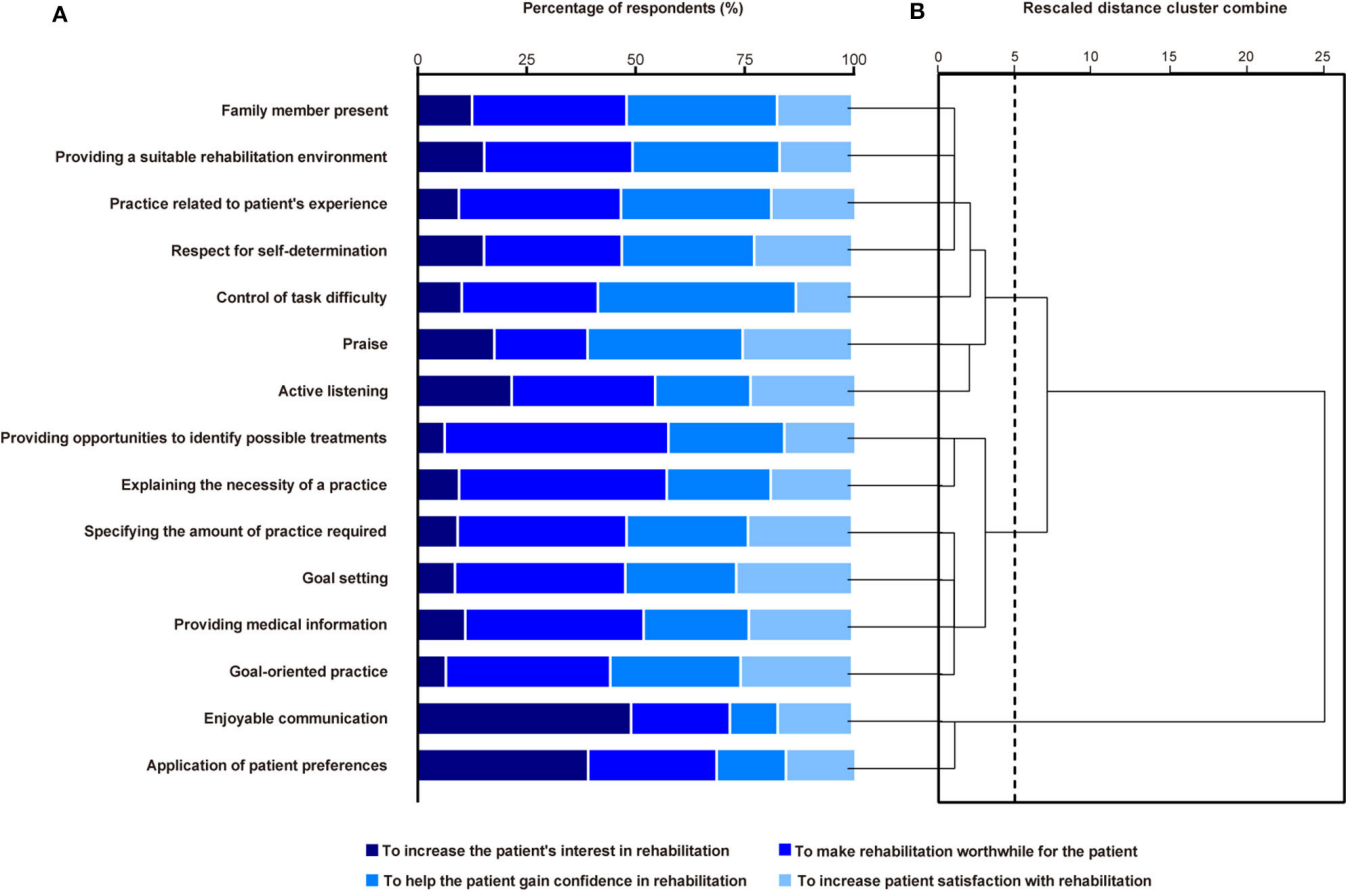
The purpose of using each motivational strategy used by at least 75% of the respondents **(A)**. The order corresponds to **(B)**. A dendrogram showing the three distinct clusters identified based on hierarchical cluster analysis via Ward's method **(B)**. The vertical dashed line indicates the optimal number of groups.

For 11 of the 15 motivational strategies that were used by at least 75% of the respondents, the highest percentage of respondents reported that they used the strategies to make rehabilitation worthwhile for their patients (51.3–31.6%) (Figure 4A). For “control of task difficulty” and “praise,” the most common purpose was to help the patient gain confidence in performing a rehabilitation task (45.4 and 35.5%, respectively). The largest proportion of respondents who used “enjoyable communication” and “application of patient preferences” reported that they used these strategies to increase the patient's interest in rehabilitation (49.3 and 39.4%, respectively).

The hierarchical cluster analysis produced three motivational strategy clusters (Figure 4B). The first cluster included seven motivational strategies, such as “family member present” and “providing a suitable rehabilitation environment.” For six of these seven strategies, more than 30% of the respondents reported that they used the strategies to help the patients gain confidence in performing a rehabilitation task. The second cluster included six motivational strategies, such as “providing opportunities to identify possible treatments” and “explaining the necessity of a practice.” Most of the respondents used these to make rehabilitation worthwhile for their patient. The third cluster comprised two strategies that the largest percentage of respondents reported having used to increase the patient's interest in rehabilitation.

### What Information Is Considered When Rehabilitation Professionals Choose Motivational Strategies?

**Figure 5:**
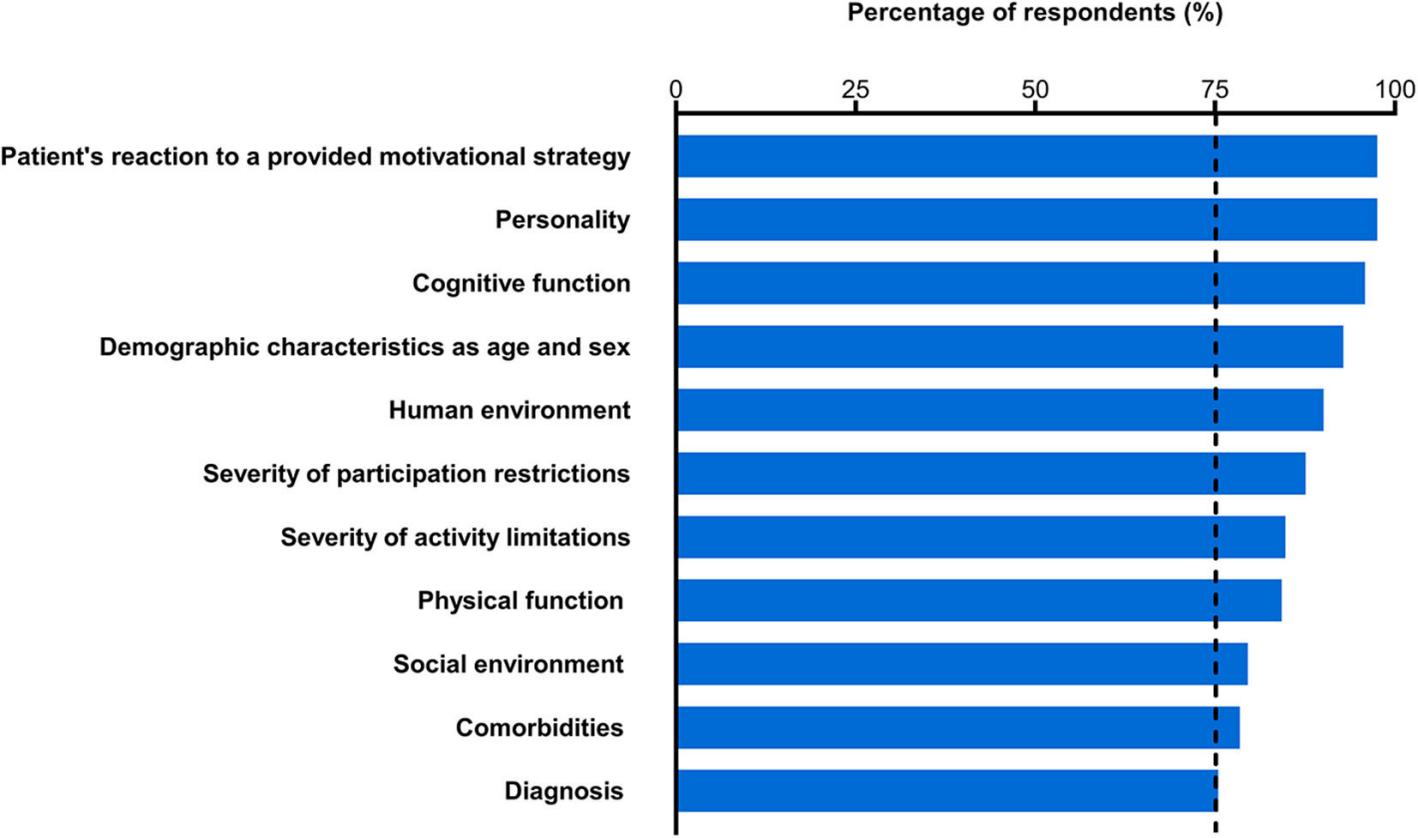
Percentage of respondents who reported considering each type of information when deciding which motivational strategy to use. The vertical dashed line represents 75% of the respondents. Information is arranged in descending order by the percentage of respondents.

Each of the presented 11 items was selected by more than 75% of respondents as information that they considered when deciding which motivational strategy to use (Figure 5). Almost all of the respondents selected “patient's reaction to a presented motivational strategy” and “personality” (97.5%), whereas the lowest percentage of respondents selected “diagnosis” (75.4%). Furthermore, approximately half of the respondents selected all of the 11 presented items (49.1%). The results for each professional category are shown in Supplemental Figure 2. More than 75% of the respondents who were physicians and nurses selected all 11 of the presented items. More than 75% of the respondents who were physical therapists and occupational therapists selected all of the items except for "diagnosis,” whereas more than 75% of the respondents who were speech-language-hearing therapists selected all of the items excluding “social environment,” “comorbidities,” and “diagnosis.”

## Discussion

As a result of the present study, we generated a comprehensive list of motivational strategies used during stroke rehabilitation in Japan based on quantitative survey data. We identified 15 motivational strategies used by more than 75% of the rehabilitation professionals who participated in the study. The results support our hypothesis that rehabilitation professionals acquire skills to motivate stroke patients via clinical experience. Furthermore, our survey generated data regarding the purpose of using each motivational strategy and the information that professionals consider when they choose motivational strategies. To our knowledge, the present study is the first comprehensive survey-based investigation of strategies used to motivate stroke patients during rehabilitation. We prepared the survey in accordance with existing guidelines (15, 16) and conducted pretesting procedures, such as expert reviews and pilot testing, to establish content and response process validity (22). We expected that these procedures would enhance the reliability and generalizability of our findings.

### Which Strategies Do Rehabilitation Professionals use to Motivate Their Patients?

Active listening is one of the core communication skills used in counseling (11, 28). A systematic review reported that counseling has a positive effect on mood in stroke patients (11, 12). Praise can induce positive mood and increase motivation to perform a motor skill, resulting in improved performance (29–31). Moreover, daily feedback with praise has been reported to be effective in improving walking speed in stroke inpatients (9). Almost all of the respondents in our study reported that they actively listened to and praised their stroke patients to increase patient motivation regarding rehabilitation.

Our results suggest that the majority of the surveyed rehabilitation professionals recognized that the communication skills of the medical professional, rehabilitation environment, setting of goals, provision of information, and the presence of family members during practice could affect patient motivation regarding rehabilitation. These findings are consistent with those of Maclean et al. (3). Communication training programs for clinicians have been shown to increase patient satisfaction, levels of motivation for goal setting and action, and health-related quality of life (32). Sonoda et al. (33) found that providing a specific rehabilitation environment for stroke inpatients increased the amount of physical activity that they engaged in during activities of daily living. Furthermore, providing information about rehabilitation and insuring that a family member is present during rehabilitation may be effective in improving a patient's mood and encouraging them to become more active (12, 34).

Respect for self-determination, control of task difficulty, and provision of a goal-oriented practice were motivational strategies that were selected by more than 75% of the respondents. Self-determination contributes to the facilitation and maintenance of intrinsic motivation (35). The difficulty of a practice task is thought to have an important impact on the effectiveness of rehabilitation, as inappropriate levels of difficulty can lead the patient to become bored or frustrated (36). Indeed, gradual increases in task difficulty and goal-oriented practice have been recommended to facilitate functional motor recovery during stroke rehabilitation (1). Through clinical practice, rehabilitation professionals may have observed the effectiveness of these motivational strategies for increasing patient adherence to rehabilitation programs.

We found that the following four strategies were reportedly used by less than half of the respondents to motivate their patients: providing practice with game properties, proposing conditions for exchange, using progress-confirming tools, and providing group rehabilitation. The reasons why these particular motivational strategies were not used remain unclear. Environmental and time constraints, professional lack of confidence regarding the practice, and lack of perceived effectiveness may prevent rehabilitation professionals from using specific motivational strategies (37). In addition, some respondents may not have selected these strategies because they did not understand the accompanying motivational effects, even if they did use these strategies in clinical practice.

### For What Purpose Do Rehabilitation Professionals use Each Motivational Strategy?

In clinical settings, rehabilitation professionals may be required to select strategies according to the cause of a patients' lack of motivation. Therefore, we examined the reasons for using each motivational strategy. For 11 of the 15 strategies used by the majority of respondents, the highest percentage of respondents reported that they used the strategy to make rehabilitation worthwhile for their patient. Lack of knowledge about the potential benefits of training may decrease patient adherence to rehabilitation (19). Therefore, professionals may emphasize patient comprehension regarding the benefits and significance of practices to motivate patients regarding rehabilitation.

Our hierarchical cluster analysis revealed three groups of motivational strategies. Strategies that center on the value of rehabilitation for patients, such as explaining the necessity of a practice and exercise, are expected to motivate patients with poor understanding regarding the benefits of rehabilitation. For patients with low confidence regarding their practice tasks, strategies focused on increasing patient confidence, such as control of task difficulty and praise, may be effective for increasing patient motivation. Moreover, engaging in enjoyable conversation and applying patient preferences to practice tasks are likely to increase patient interest and prevent patient boredom during rehabilitation. There were no strategies that were used by the majority of respondents to increase patient satisfaction with rehabilitation. However, more than a quarter of the respondents reported that they used goal setting, goal-oriented practice, and praise to increase patient satisfaction with rehabilitation. These strategies may be effective in motivating patients who are not satisfied with rehabilitation. Thus, our findings may help rehabilitation professionals to choose strategies from the list according to the cause of a patients' lack of motivation.

### What Information Is Considered When Rehabilitation Professionals Choose Motivational Strategies?

Maclean et al. (3) reported that compared with younger patients, older unmotivated stroke patients did not respond as well to encouragement. Thus, rehabilitation professionals may benefit from choosing motivational strategies according to the characteristics of each patient. Our results suggest that the majority of rehabilitation professionals choose motivational strategies based on comprehensive data regarding a patient's health condition, environmental factors, and personal factors. Patient personality type and responses to motivational strategies suggested by the professional seem to be regarded as especially essential information. Our findings may assist rehabilitation professionals in deciding which motivational strategy to use and contribute to the effective utilization of the motivational strategy list.

We also examined the participant responses by professional category (Supplemental Figure 2). We observed some differences in the responses among the different specializations. For example, while more than 75% of the physicians and nurses who participated answered that they utilized information regarding “diagnosis” when they decided which motivational strategies to use, this type of information was selected as useful by less than 75% of physical therapists, occupational therapists, and speech-language-hearing therapists. As physicians and nurses are responsible for medical management, they may be more likely to consider patient diagnosis as an important factor when choosing motivational strategies. These results may support our data indicating that different types of specialists use different strategies for motivation (Supplemental Figure 1). However, the sample of physicians and speech-language-hearing therapists in the present study was small. Thus, careful interpretation of the results by each professional category is necessary.

As mentioned above, our results showed that the different strategies were used for different purposes (Figure 4). For example, “control of task difficulty” and “praise” were most frequently used to help patients gain confidence in performing a rehabilitation task (45.4 and 35.5%, respectively), whereas the largest proportion of respondents who used “enjoyable communication” and “application of patient preferences” reported that they used these strategies to increase the patient's interest in rehabilitation (49.3 and 39.4%, respectively). However, our data suggested that many of these motivational strategies were used for one or more different purposes. For example, although the most common purpose for using “control of task difficulty” was to help patients gain confidence in performing a rehabilitation task (45.4%), the respondents also reported other purposes (10.4% to increase the patient's interest in rehabilitation; 31.2% to make rehabilitation worthwhile for the patient; 12.9% to increase patient satisfaction with rehabilitation). These different purposes for using motivational strategies might depend on the patient condition (e.g., age, comorbidities, and severity of stroke) and/or environmental factors. In the present study, we investigated the factors that are taken into consideration when rehabilitation professionals choose motivational strategies (Figure 5). However, we did not conduct concrete classifications of strategies according to the patient condition and environmental factors. Thus, future studies are needed to evaluate the motivational strategies that rehabilitation professionals use according to the patient condition and environmental factors.

## Limitations

Several limitations to this study should be mentioned. First, the actual response rate of the present survey was low (~9.8%, 407 out of 4150), and we utilized a convenience sampling method. Therefore, the results of this survey may not accurately represent the actual use of motivational strategies for stoke rehabilitation in professionals in Japan. Unfortunately, the characteristics of the nonparticipants in this survey were unavailable. We recruited participants by distributing leaflets and displaying posters, and participation was voluntarily. As a result, the nonparticipants in this study may have been less interested in the motivation of patients compared with the participants. Accordingly, the results should be interpreted with caution because the rate of use of each motivational strategy may be higher in our sample than in the general population of rehabilitation professionals. We recommend that further studies employ a stratified random sampling method to increase the range of occupations of the respondents and the generalizability of the results (38).

A secondary limitation is a weakness of generalizability of the study. Maclean et al. (3) reported that goal setting and providing medical information were thought to have a positive effect on patient motivation. In addition, Dobkin et al. (9) found that praise and positive feedback were effective for improving walking speed in patients with stroke. Thus, the present results are partly consistent with the previous qualitative (2, 3, 17, 18) and experimental reports (9, 12, 13, 34, 36) from Western countries. The advantage of this study was that it represents the first extensive survey to quantitatively investigate the actual motivational strategies used in stroke rehabilitation. However, as all of the participants were recruited in Japan, whether our findings are generalizable to rehabilitation professionals outside of Japan remains unclear. An international survey of motivational strategies used for stroke rehabilitation would improve the external validity of our findings.

Third, there may have been a perceived lack of differentiation between the motivational strategies. As our list of motivational strategies comprised specific examples about motivating a patient, some of the presented motivational strategies may have overlapped with one another (8), potentially confusing the respondents. However, we designed the motivational strategy list to show specific motivational strategies so that it would be easy to use in a clinical environment. Fourth, the responses obtained from the survey may not reflect the actual practices of the professionals. Instead, responses may have been affected by inaccurate recall or reflect the beliefs or desires of the respondents (39). However, we believe that our web-based survey carried a low participant burden and enabled more complete population coverage for sampling (39, 40). Finally, as this study had a cross-sectional design, we are unable to comment on the causal relationship between patient recovery and quantity and type of motivational strategies used. Nevertheless, our findings may be useful for examining the effect of motivational strategies on functional outcomes in the future.

## Conclusions

We generated a comprehensive and qualitative list of motivational strategies used for stroke rehabilitation in Japan, and found that our 15 motivational strategies were used by the majority of rehabilitation professionals. In addition, we obtained data regarding the purpose for using each motivational strategy and the information considered when choosing motivational strategies. These findings may enhance the effective utilization of our motivational strategy list in stroke rehabilitation.

## Data Availability Statement

The datasets generated for this study are available on request to the corresponding author.

## Ethics Statement

The studies involving human participants were reviewed and approved by Ethics committee at the Hamamatsu University School of Medicine. The patients/participants provided their written informed consent to participate in this study.

## Author Contributions

KO contributed to the study concept and design, development of the survey, acquisition of data, analysis and interpretation of data, obtaining of funding, and drafting and revising the manuscript for content. MS contributed to the development of the survey, acquisition of data, and revising of the manuscript for content. YO contributed to the study concept and design, development of the survey, and revising of the manuscript for content. ST contributed to the study concept and design, development of the survey, acquisition of data, analysis and interpretation of data, study supervision, obtaining of funding, and drafting and revising of the manuscript for content. All authors reviewed and approved the final manuscript.

## Conflict of Interest

The authors declare that the research was conducted in the absence of any commercial or financial relationships that could be construed as a potential conflict of interest.
