# Supplementary material for: Motivational Strategies for Stroke Rehabilitation: A Descriptive Cross-Sectional Study
Source: Front Neurol. 2020 Jun 10;11:553. doi: 10.3389/fneur.2020.00553 (PMC7297944; doi:10.3389/fneur.2020.00553)
Supplement: Supplementary file 1 [file Data_Sheet_1.docx]

***Supplementary Material***

## Supplementary Figures


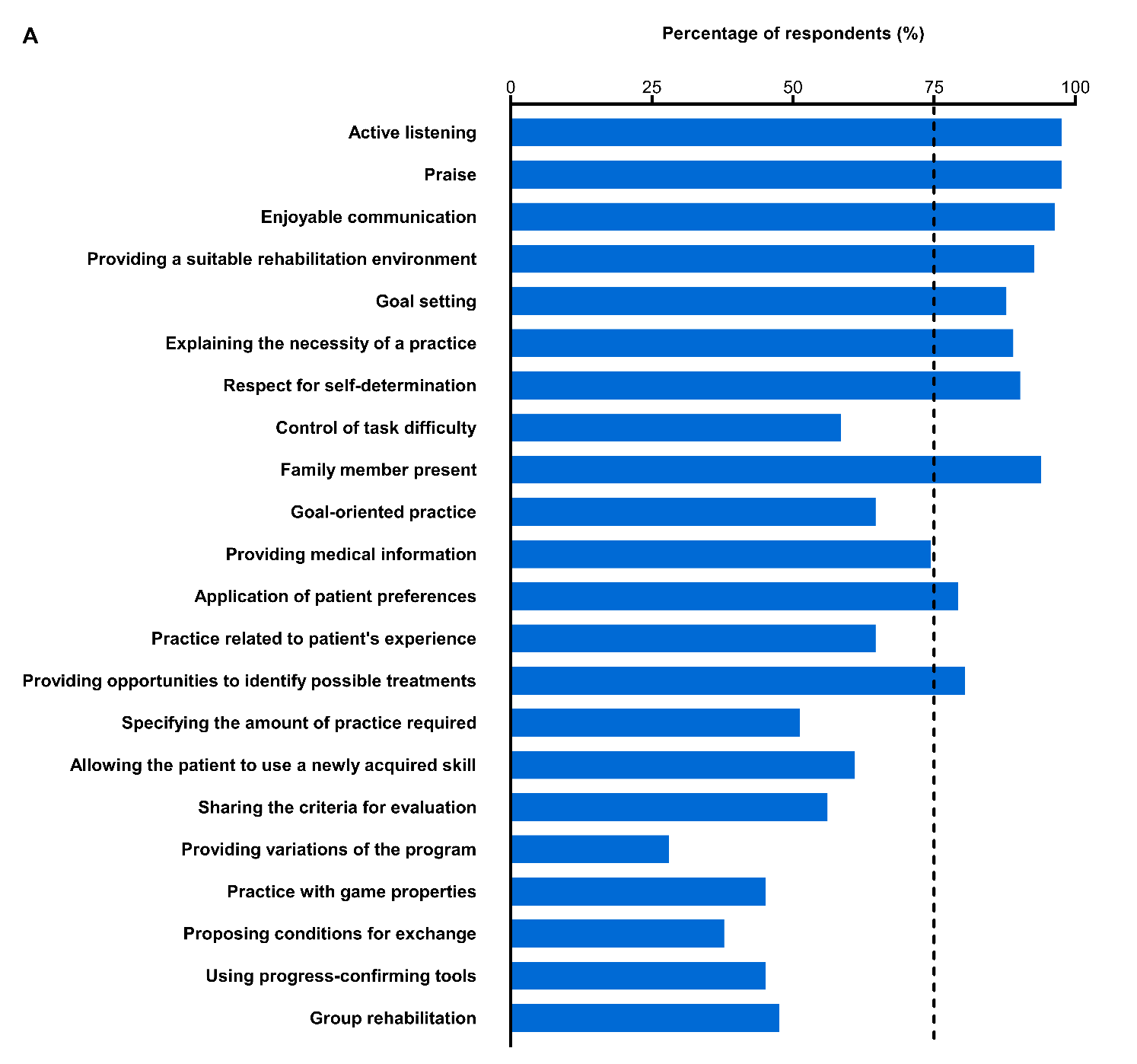


**Nurses (n = 82)**

**Figure 1A.** Percentage of nurses (n = 82) who reported using each of the presented motivational strategies. The vertical dashed line represents 75% of the respondents. The order corresponds to Figure 2.


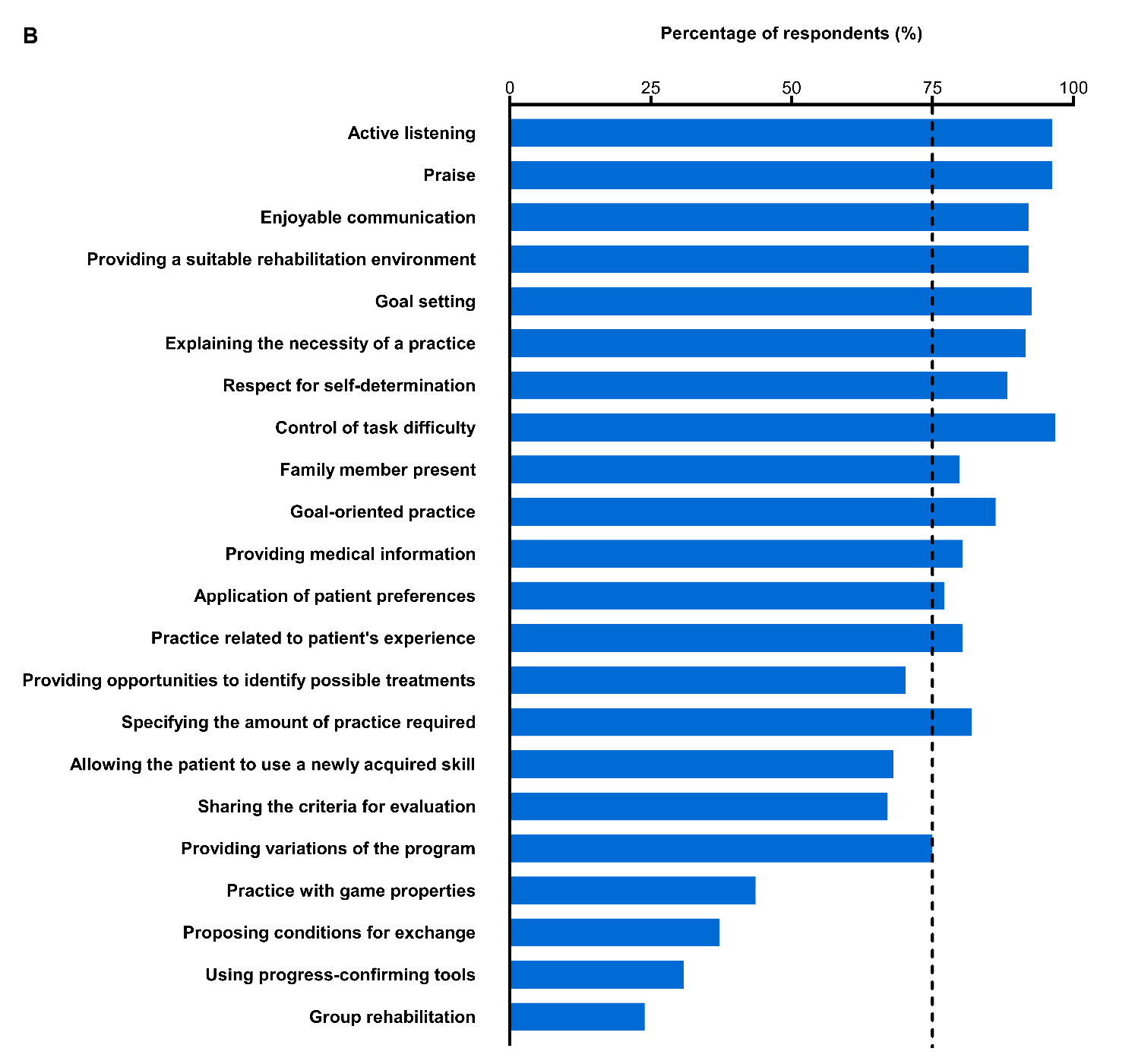


**Physical therapists (n = 185)**

**Figure 1B.** Percentage of physical therapists (n = 185) who reported using each of the presented motivational strategies. The vertical dashed line represents 75% of the respondents. The order corresponds to Figure 2.


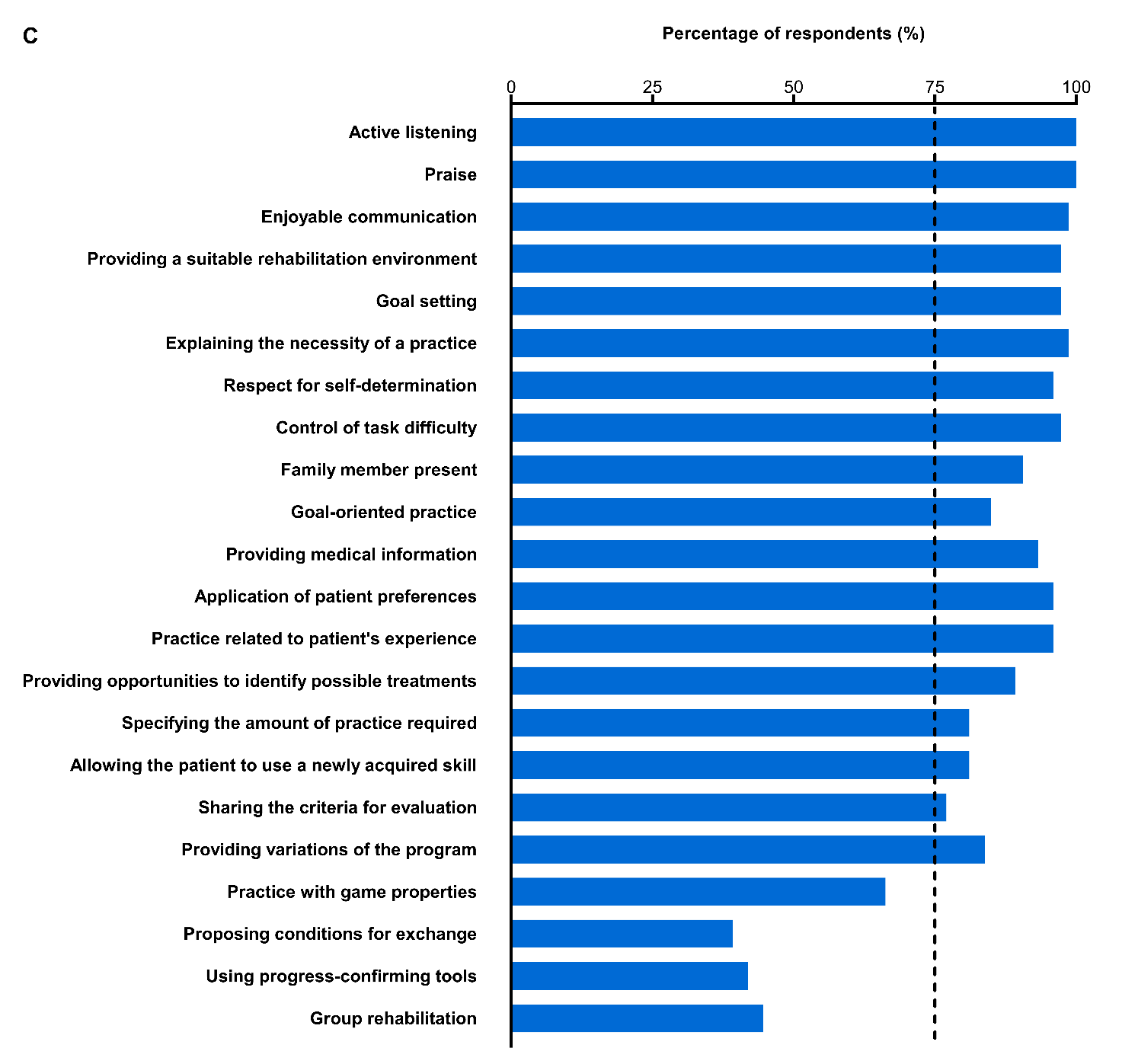


**Occupational therapists (n = 74)**

**Figure 1C.** Percentage of occupational therapists (n = 74) who reported using each of the presented motivational strategies. The vertical dashed line represents 75% of the respondents. The order corresponds to Figure 2.


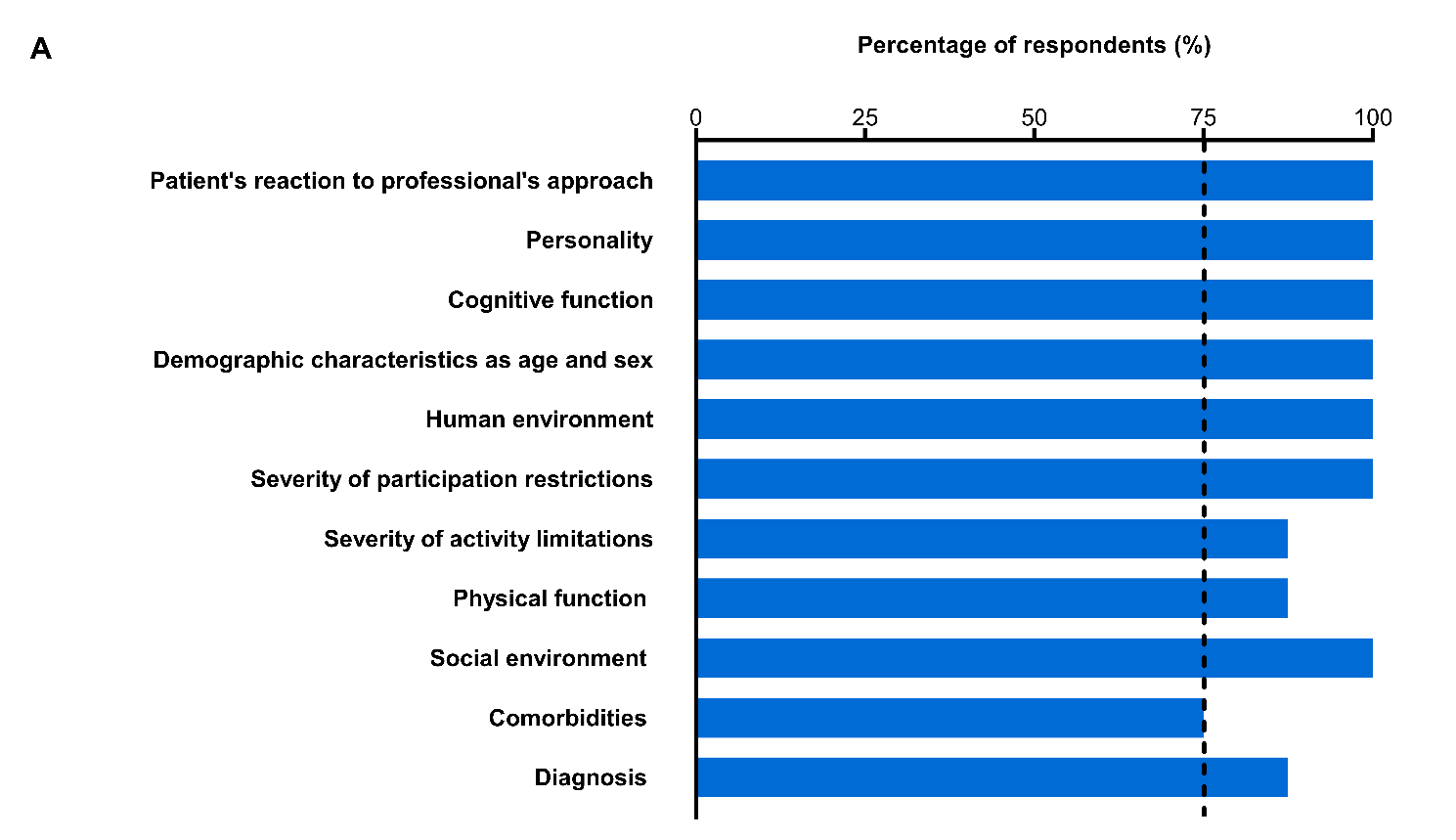


**Physicians (n = 8)**

**Figure 2A.** Percentage of physicians (n = 8) who reported considering each type of information when deciding which motivational strategy to use. The vertical dashed line represents 75% of the respondents. The order corresponds to Figure 5.


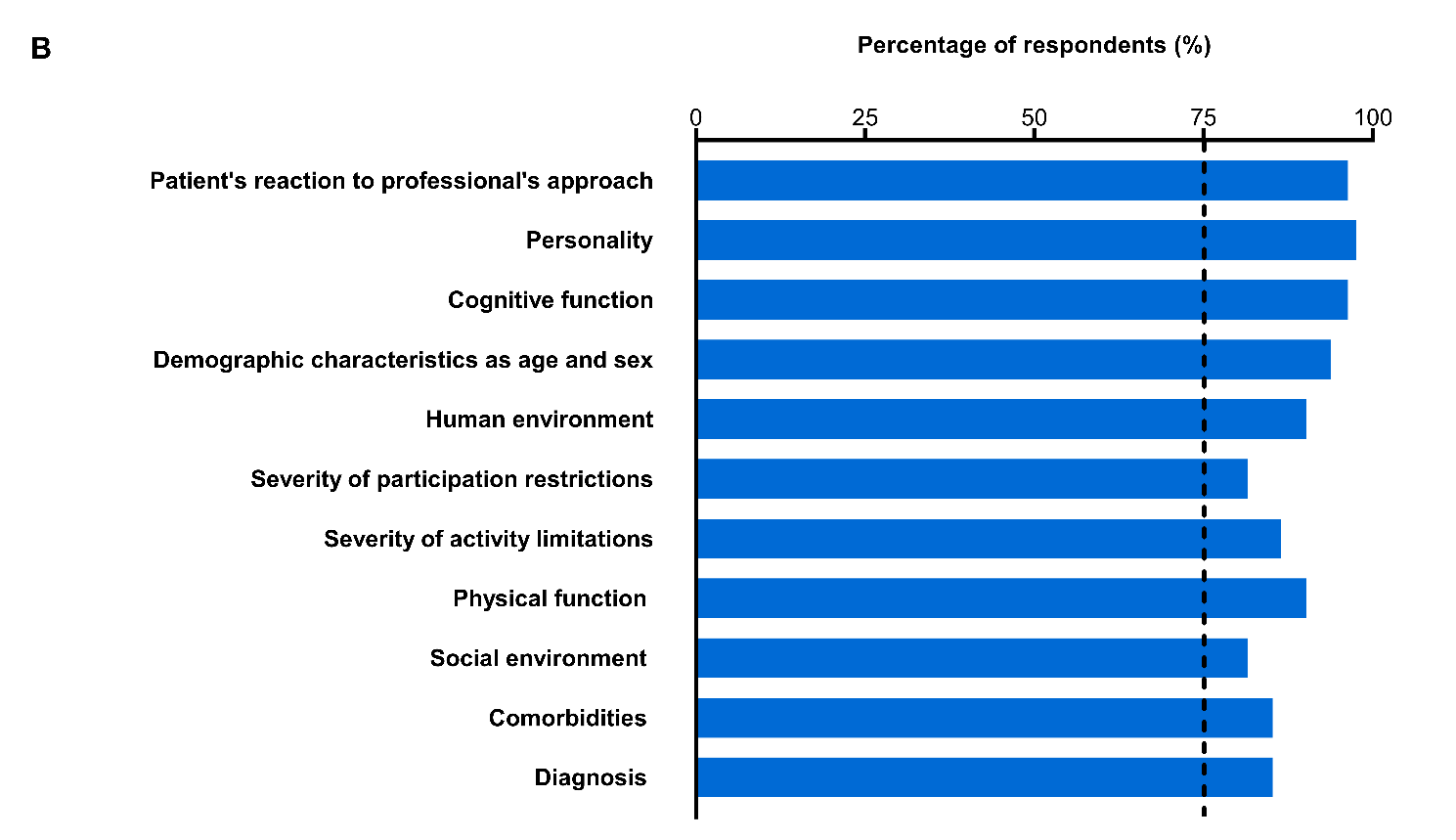


**Nurses (n = 82)**

**Figure 2B.** Percentage of nurses (n = 82) who reported considering each type of information when deciding which motivational strategy to use. The vertical dashed line represents 75% of the respondents. The order corresponds to Figure 5.


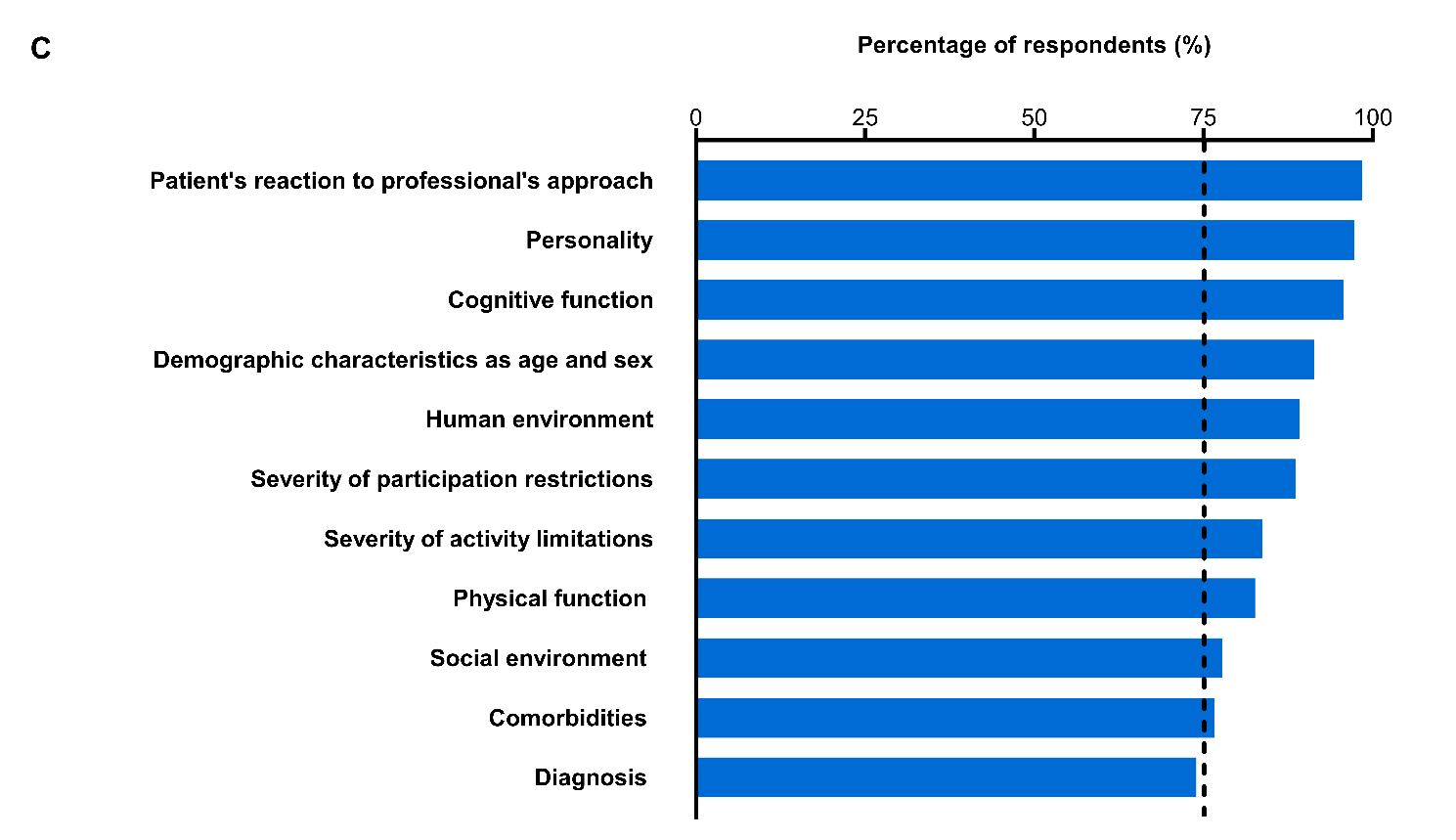


**Physical therapists (n = 185)**

**Figure 2C.** Percentage of physical therapists (n = 185) who reported considering each type of information when deciding which motivational strategy to use. The vertical dashed line represents 75% of the respondents. The order corresponds to Figure 5.


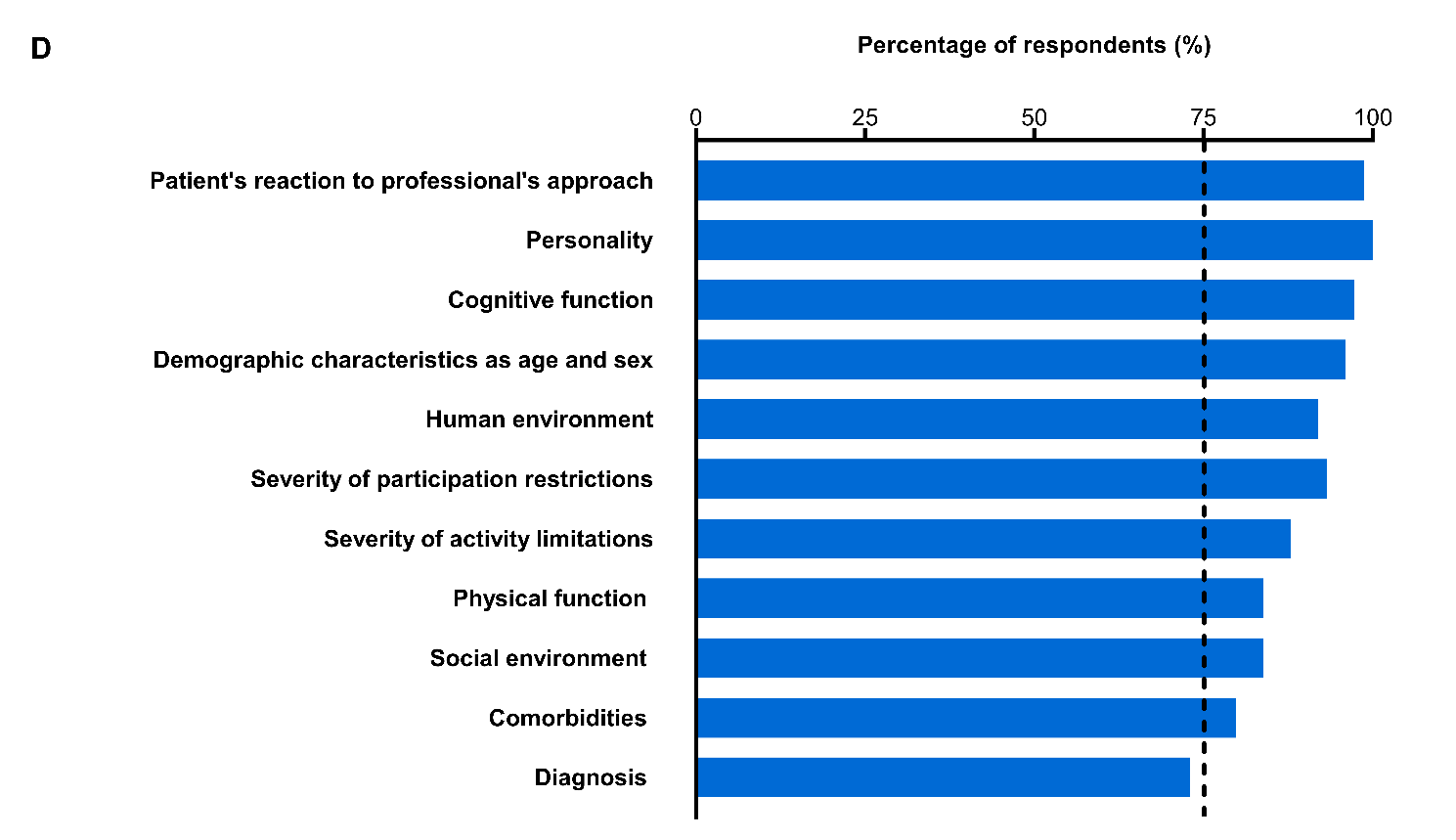


**Occupational therapists (n = 74)**

**Figure 2D.** Percentage of occupational therapists (n = 74) who reported considering each type of information when deciding which motivational strategy to use. The vertical dashed line represents 75% of the respondents. The order corresponds to Figure 5.


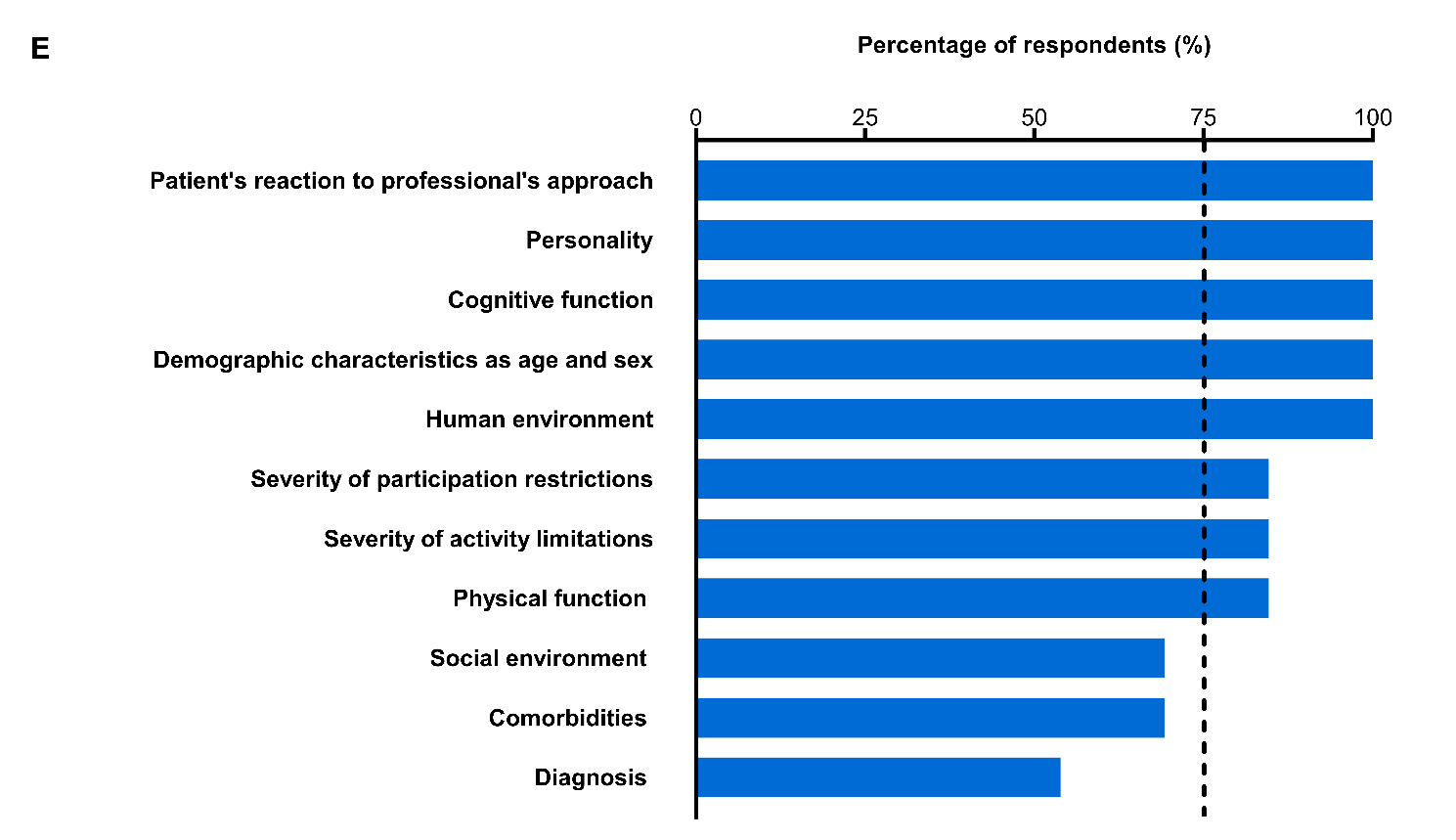


**Speech-language-hearing therapists (n = 13)**

**Figure 2E.** Percentage of speech-language-hearing therapists (n = 13) who reported considering each type of information when deciding which motivational strategy to use. The vertical dashed line represents 75% of the respondents. The order corresponds to Figure 5.

## Supplementary Table

Table 1. Additional motivational strategies proposed by the respondents

| Motivational strategy | Number of responses |
| --- | --- |
| A medical prescription | 1 |
| Do not lie to a patient | 1 |
| Practice with a patient | 1 |
| Providing feedback on the results | 3 |

*The reported motivational strategies are arranged in alphabetical order.*
